# Elp3 and Dph3 of *Schizosaccharomyces pombe* mediate cellular stress responses through tRNA^Lys^_UUU_ modifications

**DOI:** 10.1038/s41598-017-07647-1

**Published:** 2017-08-03

**Authors:** Desirée Villahermosa, Oliver Fleck

**Affiliations:** North West Cancer Research Institute, Bangor University, Deiniol Road, Brambell building, Bangor, LL57 2UW UK

## Abstract

Efficient protein synthesis in eukaryotes requires diphthamide modification of translation elongation factor eEF2 and wobble uridine modifications of tRNAs. In higher eukaryotes, these processes are important for preventing neurological and developmental defects and cancer. In this study, we used *Schizosaccharomyces pombe* as a model to analyse mutants defective in eEF2 modification (*dph1Δ*), in tRNA modifications (*elp3Δ*), or both (*dph3Δ*) for sensitivity to cytotoxic agents and thermal stress. The *dph3Δ* and *elp3Δ* mutants were sensitive to a range of drugs and had growth defects at low temperature. *dph3Δ* was epistatic with *dph1Δ* for sensitivity to hydroxyurea and methyl methanesulfonate, and with *elp3Δ* for methyl methanesulfonate and growth at 16 °C. The *dph1Δ* and *dph3Δ* deletions rescued growth defects of *elp3Δ* in response to thiabendazole and at 37 °C. Elevated tRNA^Lys^_UUU_ levels suppressed the *elp3Δ* phenotypes and some of the *dph3Δ* phenotypes, indicating that lack of tRNA^Lys^_UUU_ modifications were responsible. Furthermore, we found positive genetic interactions of *elp3Δ* and *dph3Δ* with *sty1Δ* and *atf1Δ*, indicating that Elp3/Dph3-dependent tRNA modifications are important for efficient biosynthesis of key factors required for accurate responses to cytotoxic stress conditions.

## Introduction

Dph3 plays a role in diphthamide modification of eukaryotic translation elongation factor 2 (eEF2) and in Elongator mediated modifications of tRNAs^1–5^. Dph3 is an electron donor for Dph1-Dph2, which catalyses the first step of the eEF2 modification, the transfer of 3-amino-carboxypropyl from S-adenosylmethionine to a conserved histidine of eEF2^1, 5^. In the second step, Dph5 tetra-methylates the 3-amino-carboxypropyl-histidine producing methylated diphthine. In the following step, catalysed by Dph7, de-methylation produces diphthine, which Dph6 converts to diphthamide in the final step^6, 7^. Modified eEF2 ensures error-free addition of amino acids to a growing peptide chain during translation by coordinating movement of tRNAs and mRNAs on ribosomes. In higher eukaryotes, mutations in *DPH* genes have been reported to be associated with embryonic lethality, neurological and developmental defects and cancer^8–14^.

tRNA wobble uridine modifications carried out by Elp3 as part of the Elongator complex require, like the diphthamide synthesis, S-adenosylmethionine as substrate, indicating that Dph3 also serves as an electron donor in this enzymatic reaction^2–4^. Elongator and accessory proteins catalyse 5-carbonylmethyl (cm^5^) on wobble uridines^2, 15–17^. Subsequently, cm^5^U_34_ of tRNA^Lys^_UUU_, tRNA^Glu^_UUC_, tRNA^Gln^_UUG_, tRNA^Arg^_UCU_ and tRNA^Gly^_UCC_ is methylated to 5-methoxycarbonyl-methyluridine (mcm^5^U_34_) by Trm9-Trm112^16, 18, 19^. The wobble uridines of the first three tRNA species are also thiolated by the Urm1 pathway to form 5-methoxycarbonyl-methyl-2-thiouridine (mcm^5^s^2^U_34_)^20^. cm^5^U_34_ in tRNA^Leu^_UUA_, tRNA^Val^_GUA_, tRNA^Ser^_UGA_, tRNA^Pro^_UGG_, tRNA^Thr^_UGU_ and tRNA^Ala^_UGC_ can be also modified to 5-carbamoyl-methyl-uridine (ncm^5^U_34_)^16^.

Dph3 of *Saccharomyces cerevisiae* interacts with Elp1-Elp3, although the Elongator complex is capable to form in the absence of Dph3^21^. The Elongator complex interacts with RNA polymerase II and may play a role in transcription and transcriptional silencing^4, 15, 22–24^. However, studies in *Schizosaccharomyces pombe* have indicated that transcriptional defects of *elp3Δ* mutants are rather indirectly caused by inefficient translation of transcription factors such as Atf1 and Pcr1^25^. Cells with defective Elongator lack mcm^5^U_34_ modifications at tRNA^Lys^_UUU_, tRNA^Gln^_UUG_ and tRNA^Glu^_UUC_, which results in inefficient translation of mRNAs with a high content of AAA, CAA, and GAA codons^25–27^. It has been shown that reduced protein levels in *elp3Δ* as well as sensitivity to H_2_O_2_, rapamycin and high temperature can be reversed by overexpression of tRNA^Lys^_UUU_^25, 26, 28^. Impaired tRNA modifications in humans can lead to neurological diseases and various types of cancer^29^.

While there is evidence that lack of tRNA modifications cause a reduction in the levels of a subset of proteins, we know little about how simultaneous loss of Elp3-dependent tRNA modifications and of the Dph-dependent eEF2 diphthamide modification affects cell integrity. In the present study, we have addressed this by treating *elp3Δ*, *dph3Δ* and *dph1Δ* single and double mutants of *S. pombe* with drugs that induce DNA damage and different kinds of cytotoxic stress. Genetic analyses revealed that loss of tRNA modifications were responsible for most of the phenotypes. Our data suggest that Dph3 as part of the diphthamide synthesis pathway and in conjunction with Elp3-dependent tRNA modifications ensures efficient translation of key players of stress responses such as Atf1.

## Results

### *dph3Δ* and *elp3Δ* mutants were sensitive to a range of cytotoxic agents

**Figure 1:**
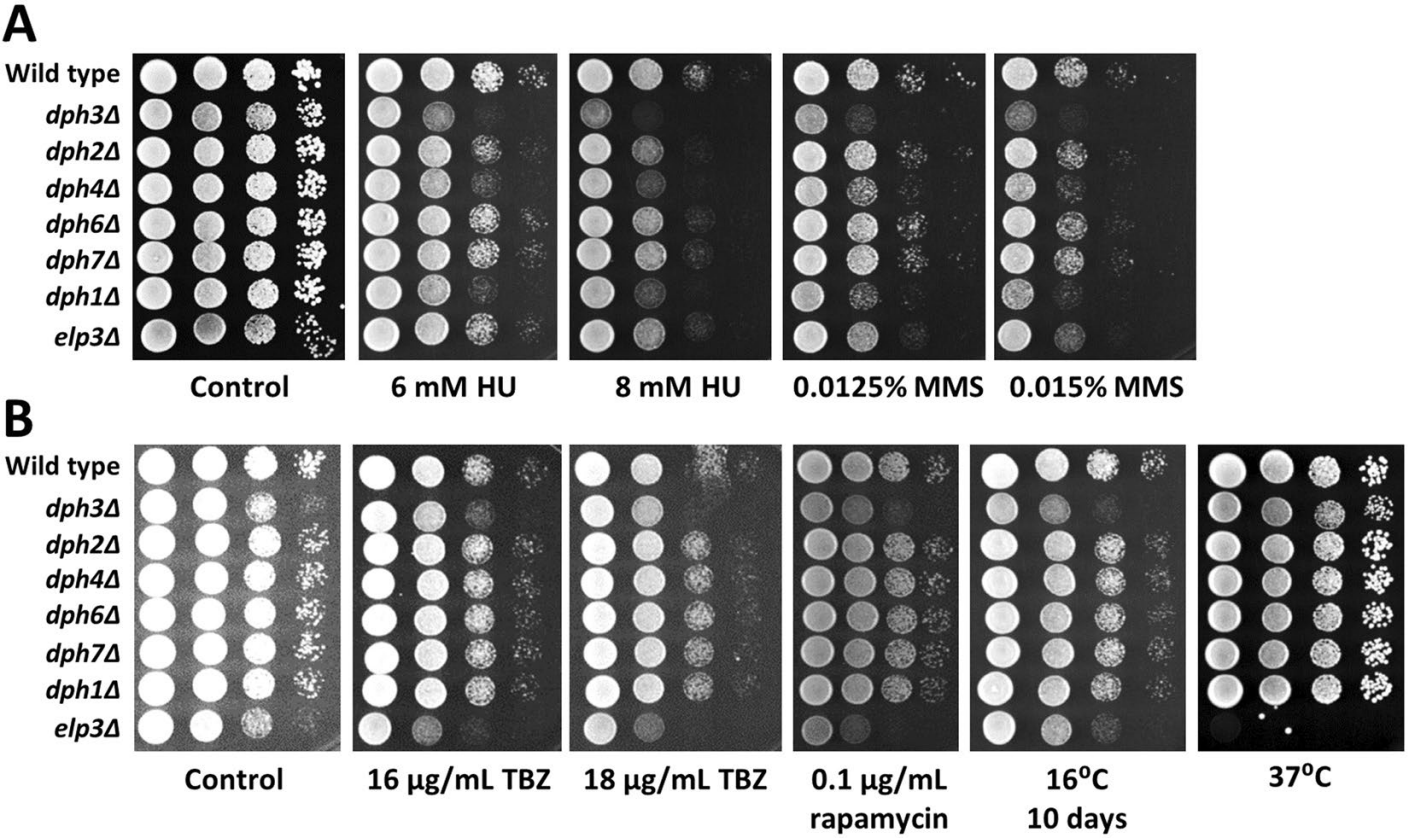
Drug and temperature sensitivity of *dphΔ* and *elp3Δ* mutants. (**A**) *dph3Δ*, *dph4Δ* and *dph1Δ* were sensitive to HU and MMS and *elp3Δ* was sensitive to MMS. Plates were incubated for three days at 30 °C. (**B**) The *dph3Δ* and *elp3Δ* mutants were sensitive to TBZ, rapamycin and low temperature. The *elp3Δ* mutant was extreme sensitive to 37 °C. Plates were incubated for two days at 30 °C, with the exceptions of one plate that was incubated for three days at 37 °C and one plate that was incubated for ten days at 16 °C. The control plate contained 0.04% DMSO.

We have previously shown that *dph3Δ* mutant cells were sensitive to hydroxyurea (HU), which depletes dNTP pools and thus stalls replication, and methyl methanesulfonate (MMS), which alkylates DNA and causes DNA breaks^30^. In this study, we were interested to know whether a defective *dph3Δ* renders cells sensitive to other cytotoxic agents or when exposed to thermal stress, and how other *dphΔ* mutants behave. We tested the *dphΔ* mutants as well as *elp3Δ* for sensitivity to HU, MMS, the microtubule depolymerising drug thiabendazole (TBZ), the TOR inhibitor rapamycin and for growth at low and high temperature. The *dph3Δ*, *dph4Δ* and *dph1Δ* mutants were sensitive to HU and MMS, while the other *dphΔ* mutants were not (Fig. 1A). The *elp3Δ* mutant was sensitive to MMS, TBZ, and rapamycin, showed reduced growth at 16 °C, and almost no growth at 37 °C. Among the *dphΔ* mutants, only *dph3Δ* was affected by rapamycin and cold (Fig. 1B). We further observed a subtle growth inhibition of *dph3Δ* in the presence of TBZ. While growth at 16 °C was rather delayed for *dph3Δ* and *elp3Δ* mutants, as they eventually caught up in growth with wild type, 37 °C appeared to kill most *elp3Δ* cells, since only a few grew to colonies when moved to 30 °C and incubated for another four days at this temperature (Supplementary Fig. S1).

In our previous study, we have found that drug resistance markers integrated at the *msh3* locus interfered with functions of the nearby located *dph3* gene^30^. In contrast, cassettes deleting *dph3* did not affect mating type switching, a process requiring Msh3. To ensure that drug and cold sensitivity of *dph3Δ* cells was not due to impaired Msh3 functions, caused by cassettes integrated at the *dph3* locus, we tested a *dph3-ATGmut* strain for sensitivity to HU, MMS, TBZ, rapamycin and for growth at 16 °C. This strain contains a mutated ATG start codon without cassette integration^30^. We found that the *dph3-ATGmut* strain was, like *dph3Δ*, sensitive to all conditions tested (Supplementary Fig. S2). Thus, the observed phenotypes were due to a defective *dph3*.

### Epistasis analysis of *dph3Δ*, *dph1Δ* and *elp3Δ*

**Figure 2:**
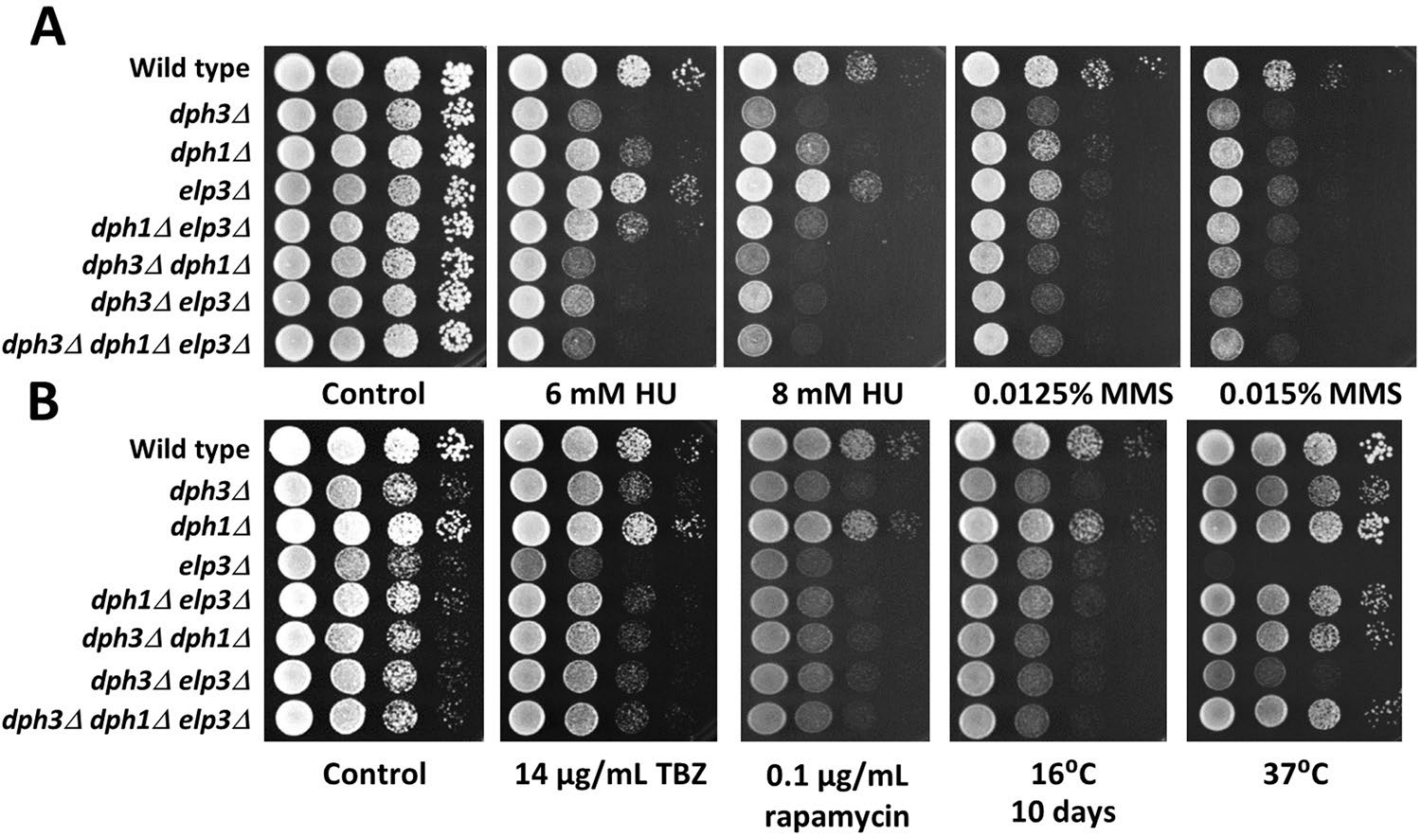
Epistasis and rescue effects with *dph3Δ*, *dph1Δ* and *elp3Δ*. (**A**) HU and MMS sensitivity. The *dph3Δ dph1Δ* and *dph3Δ dph1Δ elp3Δ* mutants showed similar sensitivity as the *dph3Δ* single mutant, indicating that Dph3 acts in the same pathways in response to HU and MMS as Dph1. The *dph1Δ elp3Δ* mutant was similarly sensitive to MMS as respective single mutants, indicating epistasis between *dph1Δ* and *elp3Δ*. The other double mutants and the triple mutant behaved like *dph3Δ*, indicating that *dph3Δ* is also epistatic with *elp3Δ*. Plates were incubated for three days at 30 °C. (**B**) *dph3Δ* and *dph1Δ* rescued the growth defect of *elp3Δ* at 37 °C and sensitivity to TBZ and rapamycin. Cold sensitivity at 16 °C appeared to be epistatic between *dph3Δ* and *elp3Δ*. The plate at 16 °C was incubated for ten days. The plate at 37 °C was incubated for three days. The other plates were incubated for two days. The control plate contained 0.033% DMSO.

**Figure 3:**
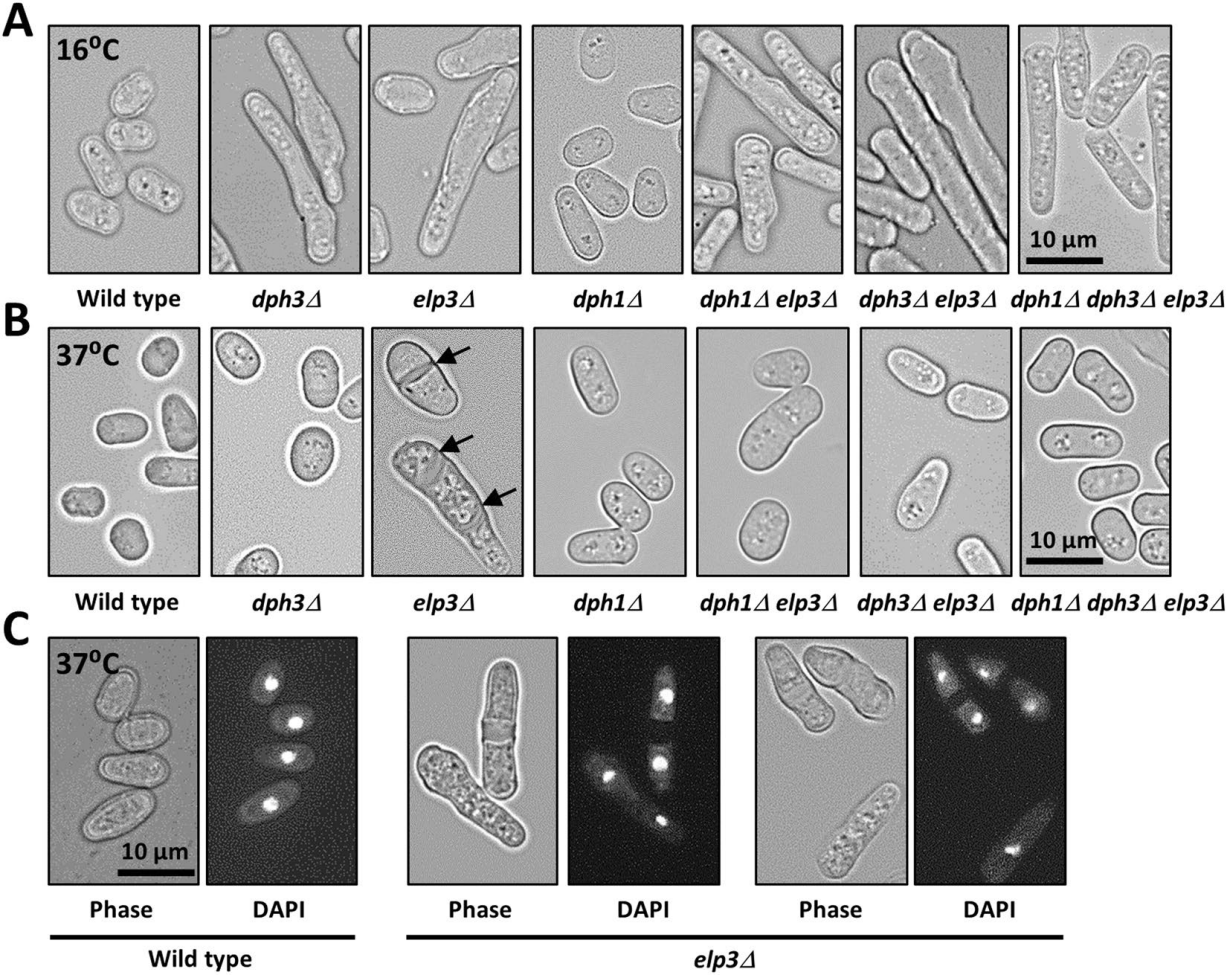
Growth at 16 °C caused elongated *dph3Δ* and *elp3Δ* cells and growth at 37 °C caused septated *elp3Δ* cells, which was rescued by *dph3Δ* and *dph1Δ*. (**A**) *dph3Δ* and *elp3Δ* cells were elongated when grown at 16 °C. (**B**) *elp3Δ* cells frequently contained one or more septa when grown at 37 °C. This phenotype was not observed when *dph3Δ* and/or *dph1Δ* were additionally deleted. (**C**) Nuclei of septated *elp3Δ* cells were not untimely torn by septa. Shown are wild type and *elp3Δ* cells in phase contrast or stained with DAPI.

Next, we performed epistasis analysis with *dph3Δ*, *dph1Δ* and *elp3Δ*. The *dph3Δ* single mutant was more sensitive to HU than *dph1Δ* and the *elp3Δ* single mutant behaved like wild type (Fig. 2A). The *dph3Δ dph1Δ* and *dph3Δ elp3Δ* double mutants and the *dph3Δ dph1Δ elp3Δ* triple mutant were similarly sensitive as the *dph3Δ* single mutant. This indicates that *dph3Δ* is epistatic with *dph1Δ* in response to HU. All three single mutants were sensitive to MMS, with *dph3Δ* being the most sensitive (Fig. 2A). The double mutants *dph3Δ dph1Δ* and *dph3Δ elp3Δ* and the triple mutant *dph3Δ dph1Δ elp3Δ* exhibited similar MMS sensitivity as the *dph3Δ* single mutant. The *dph1Δ elp3Δ* double mutant was slightly less sensitive, with a survival in the range of the *dph1Δ* and *elp3Δ* single mutants. Thus, *dph3Δ*, *dph1Δ* and *elp3Δ* were epistatic for MMS sensitivity. We further tested sensitivity to TBZ and rapamycin and growth at 16 °C and 37 °C. When treated with rapamycin or grown at 16 °C, *dph3Δ* and *elp3Δ*, but not *dph1Δ*, were affected (Fig. 2B). The *dph3Δ elp3Δ* double mutant showed a similar level of reduced growth at 16 °C as the *dph3Δ* and *elp3Δ* single mutants, indicating epistasis between these two deletions. Cells of *dph3Δ* and *elp3Δ* single, double and triple mutants, but not of the *dph1Δ* single mutant or of wild type, were elongated when grown at 16 °C (Fig. 3A). We further found that *dph3Δ* and *dph1Δ* rescued the growth defects of *elp3Δ* at 37 °C and in the presence of TBZ, and more subtly, sensitivity to rapamycin (Fig. 2B). The *elp3Δ* mutant grown at 37 °C had an increased number of cells with septa. 24% of the cells contained one septum, which was in some cells misplaced or relatively thick and 5% of the *elp3Δ* cells contained two or more septa (Table 1, Fig. 3B). Such a phenotype was not observed in wild type, *dph1Δ* or *dph3Δ* cells. When grown at 30 °C, the frequency of septated *elp3Δ* cells was as low as for wild type and the other mutant strains (Table 1). The nuclei of septated *elp3Δ* cells appeared to be like those of wild type cells without any abnormalities detectable, such as a “cut” (cell untimely torn) phenotype or unequal distribution of the genetic material (Fig. 3C). The occurrence of septated *elp3Δ* cells at 37 °C was reduced to the wild type level when *dph3* and/or *dph1* were additionally deleted (Table 1 and Fig. 3B). Our data indicate that the presence of Dph3 and Dph1 in the *elp3Δ* single mutant is detrimental to the cells when grown at 37 °C or exposed to TBZ.

**Table 1:** Increased number of septated *elp3Δ* cells at 37 °C.

Relevant genotype	Temperature							
	30 °C		37 °C					
	Number of septa per cell							
	0	1	0	p-value	1	p-value	≥2	p-value
Wild type	98 (1.2)	2 (1.2)	98 (1)		2 (1)		0	
*dph3Δ*	99 (1.2)	1 (1.2)	99 (1)		1 (1)		0	
*elp3Δ*	97 (3.5)	3 (3.5)	71 (3.3)	7.1 × 10^−7^	24 (4.7)	5.2 × 10^−5^	5 (2.6)	0.0098
*dph3Δ elp3Δ*	99 (1.5)	1 (1.5)	99 (1)	5.4 × 10^−7^	1 (1)	3.8 × 10^−5^	0	0.0098
*dph1Δ*	99 (0.6)	1 (0.6)	98 (1.7)		2 (1.7)		0	
*dph1Δ elp3Δ*	99 (0.6)	1 (0.6)	99 (1.9)	7.9 × 10^−8^	1 (1.9)	7.5 × 10^−6^	0	0.0035
*dph3Δ dph1Δ elp3Δ*	99 (1.0)	1 (1)	99 (1.7)	7.7 × 10^−8^	1 (0.6)	4.3 × 10^−6^	0	0.0035

Numbers in percent with standard deviations in parentheses.

p-values were calculated using the two-tailed student’s T-test. p-values for *elp3Δ* calculated by comparison with wild type; p-values for *dph3Δ elp3Δ*, *dph1Δ elp3Δ* and *dph3Δ dph1Δ elp3Δ* calculated by comparison with *elp3Δ*.

### Rescue of drug and temperature sensitivity by elevated tRNA^Lys^_UUU_ levels

**Figure 4:**
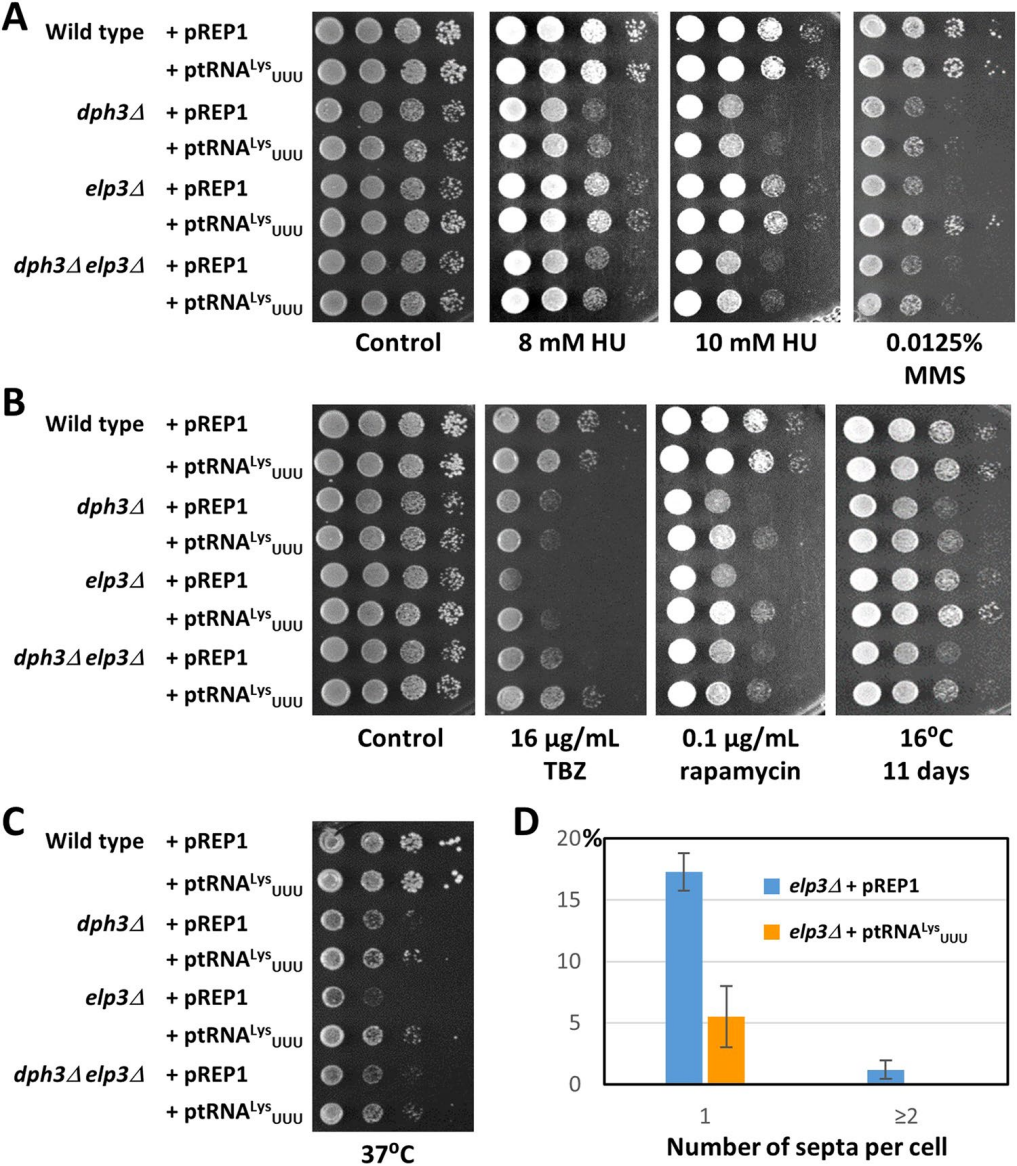
Rescue of *dph3Δ* and *elp3Δ* phenotypes by increased tRNA^Lys^_UUU_ levels. Strains containing either the vector pREP1 or a plasmid expressing the gene for tRNA^Lys^_UUU_ (*SPBTRNALYS.06*) were spotted on minimal medium with the indicated drugs and 5 μg/mL thiamine to repress the *nmt* promoter of pREP1. (**A**) HU and MMS sensitivity. (**B**) TBZ and rapamycin sensitivity and growth at 16 °C. The control plate contained 0.04% DMSO. (**C**) Growth at 37 °C. Excess of tRNA^Lys^_UUU_ was able to rescue sensitivity of *elp3Δ* to MMS, TBZ, rapamycin and growth at 16 °C and 37 °C. In the case of *dph3Δ*, sensitivity to rapamycin and growth at 16 °C and 37 °C was rescued. Plates were incubated for three days, except the plate containing rapamycin and the plate at 16 °C, which were incubated for two and eleven days, respectively. (**D**) Increased tRNA^Lys^_UUU_ levels lowered the number of septated *elp3Δ* cells at 37 °C. 5.5% of *elp3Δ* + ptRNA^Lys^_UUU_ cells contained one septum, whereas 17.3% of *elp3Δ* + pREP1 cells had one septum (p = 0.002). 1.2% of *elp3Δ* + pREP1 cells and none of *elp3Δ* + ptRNA^Lys^_UUU_ cells had two or more septa (p = 0.057; not significant). Columns represent averages of three independent counts of 100 or 200 cells with standard deviations.

It has been previously shown that an excess of unmodified tRNA^Lys^_UUU_ encoded on a multi-copy plasmid suppressed sensitivity of *elp3Δ* to H_2_O_2_ and growth inhibition at 36 °C^25, 26^. We therefore tested whether elevated tRNA^Lys^_UUU_ levels could complement the *dph3Δ* and *elp3Δ* phenotypes described in our study. We observed for *dph3Δ* that reduced growth at 16 °C and 37 °C and sensitivity to rapamycin was suppressed, whilst sensitivity to HU and TBZ remained largely unaffected (Fig. 4). A small effect was found in response to MMS. In the case of *elp3Δ*, elevated tRNA^Lys^_UUU_ rescued all phenotypes, i.e. sensitivity to MMS, TBZ and rapamycin and growth inhibition at 16 °C and 37 °C. The *dph3Δ elp3Δ* double mutant containing the control plasmid pREP1 was less sensitive to TBZ than *elp3Δ* + pREP1, indicating rescue of *elp3Δ* sensitivity by *dph3Δ*, consistent with our previous findings (Fig. 2). *dph3Δ elp3Δ* + ptRNA^Lys^_UUU_ was even more resistant to TBZ than *dph3Δ elp3Δ* + pREP1, and also more resistant than *elp3Δ* + ptRNA^Lys^_UUU_ (Fig. 4B). Thus, tRNA^Lys^_UUU_ complemented TBZ sensitivity of *elp3Δ* independently of *dph3Δ*. A rescue effect was also observed for the rapamycin treated double mutant, although here, the effects of *dph3Δ* and ptRNA^Lys^_UUU_ may not be independently of each other.

We noticed that at 37 °C *dph3Δ* grew significantly better on complex medium than on minimal medium and that the opposite was true for *elp3Δ* (Figs 1, 2 and 4). We observed an elevated number of septated cells of *elp3Δ* transformed with pREP1 (Fig. 4D). However, it was significantly lower than for *elp3Δ* grown on complex medium (Table 1) (18.5 ± 2.3% versus 29.5 ± 2.5%; p = 0.002). The presence of high tRNA^Lys^_UUU_ levels in *elp3Δ* cells reduced the occurrence of septated cells to 5.5 ± 2.5% (p = 0.0027) (Fig. 4D). Thus, growth inhibition and septation at 37 °C either appeared to be dependent on the medium or was due to the presence of plasmids.

To test this and to demonstrate that the rescue effects were indeed due to the presence of ptRNA^Lys^_UUU_, we isolated *elp3Δ* mutants that had lost their plasmids (pREP1 or ptRNA^Lys^_UUU_) by culturing them in complex medium and isolating *leu1-32* auxotrophic strains. Such strains were spotted on minimal medium supplemented with leucine and were grown at either 30 °C or 37 °C (Supplementary Fig. S3A). At 37 °C, all *elp3Δ* mutants showed growth inhibition and an increased number of septated cells when compared to wild type (Supplementary Table S1). We conclude that the presence of ptRNA^Lys^_UUU_ in *elp3Δ* cells rescued temperature sensitivity and prevented accumulation of septated cells and that loss of the plasmid rendered the mutant sensitive again and caused a higher number of septated cells. In addition, we compared growth of *dph3Δ* and *elp3Δ* transformed with the vector pREP1 at 37 °C with untransformed mutant strains and did not found a relevant difference (Supplementary Fig. S3B).

### The ribosomal *rpl42-P56Q* mutation caused MMS and HU sensitivity, which was suppressed by *dph3Δ* and *elp3Δ*

**Figure 5:**
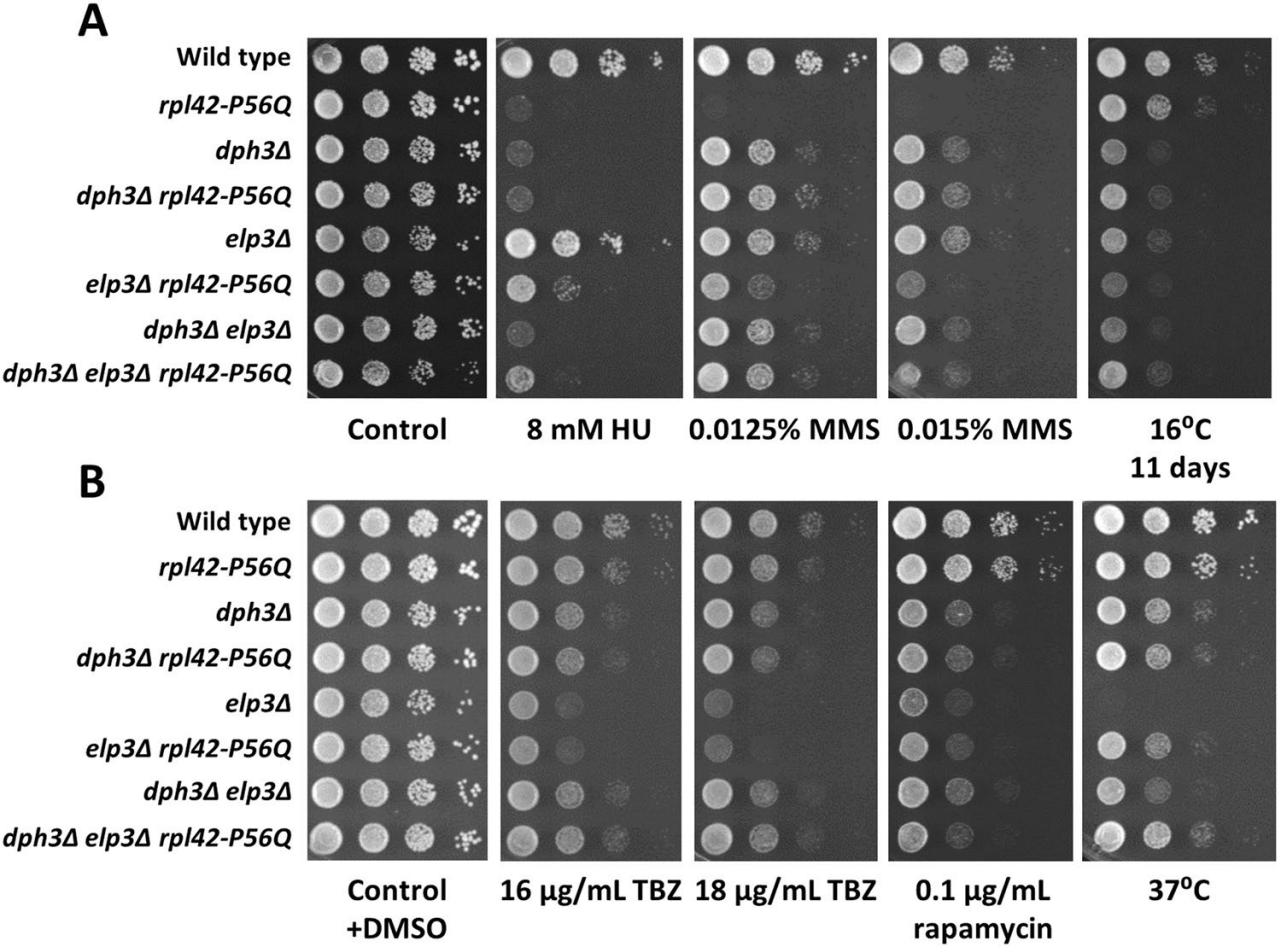
Genetic interactions of *dph3Δ* and *elp3Δ* with *rpl42-P56Q*. (**A**) *rpl42-P56Q* was sensitive to HU and MMS. MMS sensitivity was rescued by *dph3Δ* and *elp3Δ*. HU sensitivity was rescued by *elp3Δ*. Here, the genetic relationship between *dph3Δ* and *rpl42-P56Q* is less clear. The mutations were either epistatic or *dph3Δ* showed a minor suppression effect. (**B**) The growth defect of *elp3Δ* at 37 °C was independently rescued by *rpl42-P56Q* and *dph3Δ*.

The *rpl42-P56Q* mutation of the 60 S ribosomal protein L42 interferes with translation elongation, likely through altered contacts with tRNAs^31^. We used this mutation to test genetic interaction with *dph3Δ* and *elp3Δ*. The *rpl42-P56Q* mutant itself was sensitive to HU and MMS, but not to TBZ, rapamycin or thermal stress (Fig. 5). MMS sensitivity of *rpl42-P56Q* was rescued by *dph3Δ* and *elp3Δ*. We further observed that *elp3Δ* complemented HU sensitivity of *rpl42-P56Q*. In the case of *dph3Δ*, we found either a weak suppression or epistasis with *rpl42-P56Q*, as the double mutant exhibited sensitivity like *dph3Δ*, and close to *rpl42-P56Q* (Fig. 5A). On the other hand, *rpl42-P56Q* rescued temperature sensitivity of *elp3Δ*. Since the *dph3Δ elp3Δ rpl42-P56Q* triple mutant grew better at 37 °C than the *dph3Δ elp3Δ* and *elp3Δ rpl42-P56Q* double mutants, suppression by *rpl42-P56Q* likely occurred independently of *dph3Δ* mediated rescue.

### Global protein levels were not significantly affected in *elp3Δ* and *dph3Δ* mutants

Translation of mRNAs is affected in *elp3Δ* and *dph3Δ* due to lack of tRNA modifications and in the latter mutant also due to lack of the diphthamide modification of eEF2^1–5, 16^. We therefore tested global protein levels of *dph3Δ* and *elp3Δ* single and double mutants grown at 30 °C and 37 °C. We could not detect any significant differences to wild type at either temperature (Supplementary Fig. S4). Thus, under the conditions tested general protein synthesis appeared to be not affected in *dph3Δ* and *elp3Δ* mutants.

### *elp3Δ* and *dph3Δ* showed positive genetic interactions with the stress response mutants *sty1Δ* and *atf1Δ*

**Figure 6:**
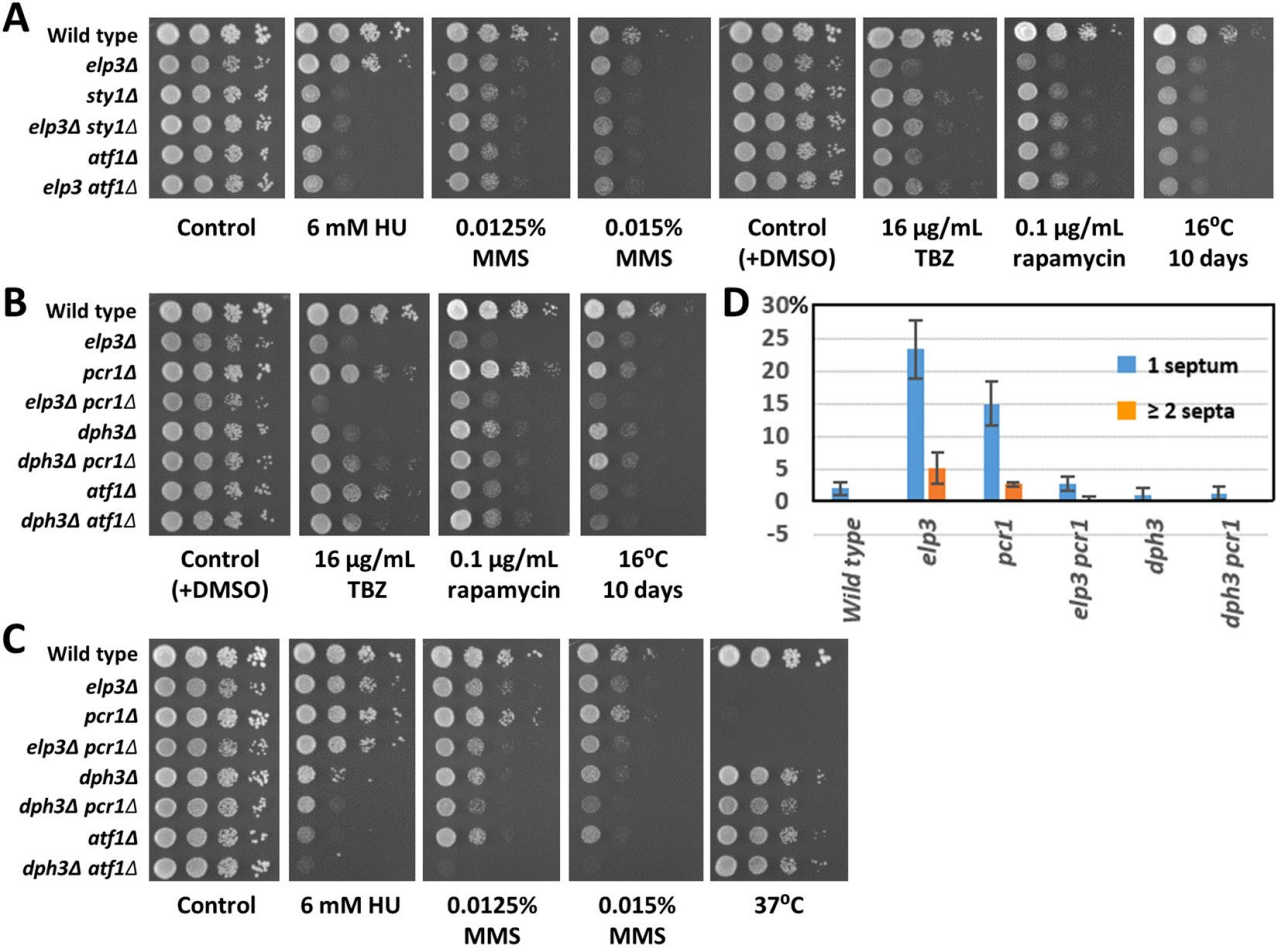
Genetic interactions of *elp3Δ* and *dph3Δ* with stress response mutations. (**A**) *sty1Δ* and *atf1Δ* showed positive genetic interactions (epistasis or weak rescue) with *elp3Δ* for MMS, TBZ, rapamycin and cold sensitivity. (**B**) *dph3Δ* was epistatic with *atf1Δ* for rapamycin and cold sensitivity. For TBZ a mild rescue was observed. The *pcr1Δ* mutant was cold sensitive independently of *elp3Δ*. (**C**) A *dph3Δ atf1Δ* double mutant was more sensitive to HU and MMS than respective single mutants, indicating independent functions of Dph3 and Atf1 in response to these drugs. Growth of *pcr1Δ* at 37 °C was inhibited, but rescued by *dph3Δ*. (**D**) The *pcr1Δ* and *elp3Δ* single mutants, but not the *elp3Δ pcr1Δ* and *dph3Δ pcr1Δ* double mutants, frequently had septated cells. Columns represent averages with standard deviations of at least three independent experiments, each with 200 counted cells. Values for wild type and *dph3Δ* were taken from Table 1. p-values for the occurrence of septated cells versus wild type were 2.2 × 10^−7^ for *elp3Δ* and 0.001 for *pcr1Δ*. p-values of *elp3Δ pcr1Δ* were 3.3 × 10^−8^ and 3.1 × 10^−4^ versus *elp3Δ* and *pcr1Δ*, respectively. The p-value of *dph3Δ pcr1Δ* versus *pcr1Δ* was 8.9 × 10^−4^.

The *S. pombe* transcription factors Atf1 and Pcr1 have been identified as translational targets of Elp3^25^. Atf1 and Pcr1 protein levels were increased in H_2_O_2_ treated wild type cells, but remained low in an *elp3Δ* mutant. Atf1-Pcr1 is activated by the MAP kinase Sty1 in response to cellular stress^32^. Here, we tested sensitivity of *sty1Δ*, *atf1Δ* and *pcr1Δ* mutants to cytotoxic stress and performed epistasis analysis with *elp3Δ* and *dph3Δ*. The rationale was that if a double mutant was not more sensitive than the most sensitive single mutant, then the phenotypes of the *elp3Δ* and *dph3Δ* single mutants were (at least partially) due to low protein levels of the transcription factors Atf1 or Pcr1. In *sty1Δ* mutants, Atf1 cannot be activated by phosphorylation, display a low protein level, and thus, transcriptional activation of stress response genes by Atf1 is diminished^32^. We have found that *sty1Δ* and *atf1Δ* mutants were sensitive to HU, MMS, TBZ, and rapamycin and exhibited slow growth at 16 °C (Fig. 6A). MMS sensitivity and growth at 16 °C of the *elp3Δ sty1Δ* and *elp3Δ atf1Δ* double mutants was comparable to that of the single mutants, indicating epistasis of *elp3Δ* with *sty1Δ* and *atf1Δ*. In the cases of TBZ and rapamycin, the double mutants showed sensitivity similar to the *sty1Δ* and *atf1Δ* single mutants, which were less sensitive than *elp3Δ*. This difference could be due to detrimental effects of inefficient synthesis of Atf1 in the *elp3Δ* single mutant, which, for example, could lead to misfolded Atf1 protein, which interferes with cellular functions. For HU sensitivity, we did not obtain information about genetic interactions, since the *elp3Δ* single mutant was not sensitive.

*dph3Δ* was epistatic with *atf1Δ* for rapamycin sensitivity and for growth inhibition at 16 °C (Fig. 6B), but not for HU or MMS, where the double mutant was clearly more sensitive than either single mutant (Fig. 6C). Thus, we found epistasis for MMS survival between *elp3Δ* and *dph3Δ* (Fig. 2A), between *elp3Δ* and *atf1Δ* (Fig. 6A), but not between *dph3Δ* and *atf1Δ* (Fig. 6C). The simplest explanation of the observed genetic interactions is that Dph3 and Atf1 likely have Elp3-independent functions in response to MMS, which are also independent to each other. This is consistent with the observations that the *dph3Δ* and *atf1Δ* single mutants were more sensitive to MMS than *elp3Δ* (Fig. 6C). For TBZ, we found a mild rescue effect of *dph3Δ* by *atf1Δ* (Fig. 6B).

The *pcr1Δ* single mutant was not sensitive to any of the cytotoxic agents tested (Fig. 6B,C), but growth was inhibited at 37 °C, which was rescued by additionally deleting *dph3* (Fig. 6C). Like *elp3Δ*, the *pcr1Δ* single mutant, but not the *elp3Δ pcr1Δ* and *dph3Δ pcr1Δ* double mutants, had an increased number of septated cells (Fig. 6D). The *pcr1Δ* mutant also grew slowly at 16 °C (Fig. 6B). Since the *elp3Δ pcr1Δ* double mutant grew slower than either single mutant, cold sensitivity of *elp3Δ* was independent of *pcr1Δ*, and thus likely not due to low Pcr1 levels.

Taken together, survival of cells treated with HU, MMS, TBZ or rapamycin or when grown at 16 °C required the stress response factors Atf1 and Sty1. Since Atf1 has been shown to be regulated on the translational level by Elp3 via tRNA modifications^25^, and since the *elp3Δ atf1Δ* and the *elp3Δ sty1Δ* double mutants revealed epistasis, it is likely that phenotypes of *elp3Δ* were -to some extent- due to low Atf1 levels. This is also true for *dph3Δ* for TBZ, rapamycin and cold sensitivity.

## Discussion

**Figure 7:**
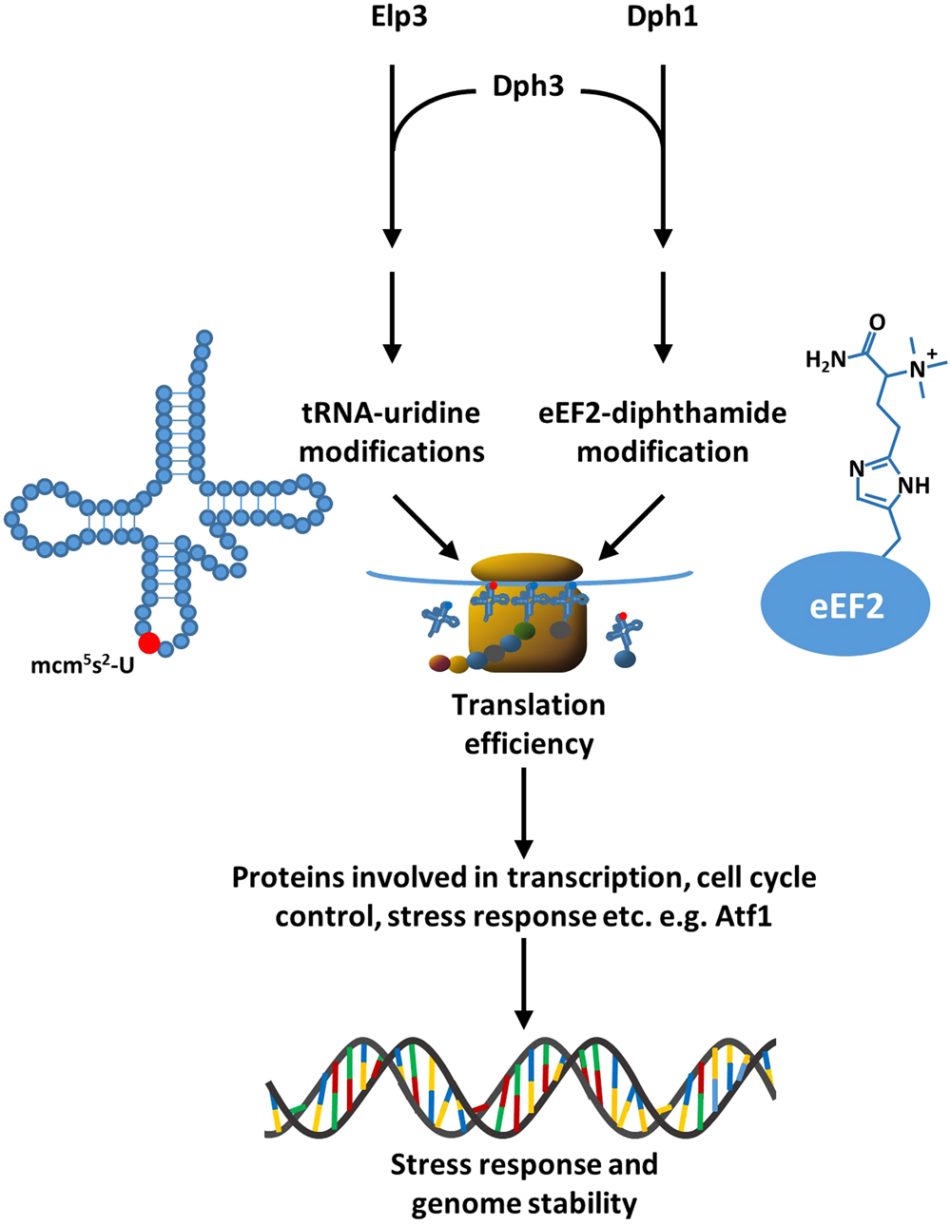
Model for the contribution of Dph3 in stress responses. Dph3 is supposed to be an electron donor for the Elp3-containing Elongator complex for modifications of wobble uridines in tRNA anticodons and for Dph1-Dph2 to initiate the first step of diphthamide modification of eEF2. Both modifications are required for efficient translation of mRNAs to proteins at ribosomes to produce key proteins involved in transcription, stress and DNA damage responses and other pathways that ensure accurate responses to cytotoxic agents and genome stability. One identified factor with reduced protein levels in *elp3Δ* background is Atf1^25^. An *atf1Δ* mutant showed positive genetic interactions with *elp3Δ* and *dph3Δ* (Fig. 6).

In the present study, we have analysed drug and thermal sensitivity caused by deletions of *dph* genes and *elp3* in *S. pombe*. Dph proteins are required for diphthamide modification of eEF2^7^. Elp3 is part of the Elongator complex, which is required for wobble uridine modifications of tRNAs^2, 3, 15, 28^. Dph3 participates in both types of modifications^3, 7, 21^ (Fig. 7). We have performed epistasis analysis and tested rescue effects mediated by elevated tRNA^Lys^_UUU_ levels, by a ribosomal *rpl42-P56Q* mutation, and by *dph3Δ* and *elp3Δ*. In addition, genetic interactions of *dph3Δ* and *elp3Δ* with *sty1Δ*, *atf1Δ* and *pcr1Δ* have been analysed. The results are summarised in Tables 2 and 3.

**Table 2:** Summary for drug and temperature sensitivity of *dphΔ* and *elp3Δ* mutants.

Mutant	HU	MMS	TBZ	Rapamycin	16 °C	37 °C
*dph1Δ*	S	S	+	+	+	+
*dph2Δ*	+	+	+	+	+	+
*dph3Δ*	S	S	S	S	S	+/S
*dph4Δ*	S	S	+	+	+	+
*dph6Δ*	+	+	+	+	+	+
*dph7Δ*	+	+	+	+	+	+
*elp3Δ*	+	S	S	S	S	S

+ Indicates no difference to wild type. S indicates sensitivity. +/S indicates no significant difference of *dph3Δ* to wild type on complex medium, but growth inhibition on minimal medium. Note that *elp3Δ* was the only other mutant that we tested on minimal medium (Fig. 4).

**Table 3:** Summary of genetic interactions.

	*dph3Δ*			*elp3Δ*	ptRNA^Lys^_UUU_		*rpl42-P56Q*	
	*elp3Δ*	*dph1Δ*	*atf1Δ*	*atf1Δ*/*sty1Δ*	*elp3Δ*	*dph3Δ*	*elp3Δ*	*dph3Δ*
HU		E	S				R^b^	(R)^b^
MMS	E	E	S	E	R	(R)	R^b^	R^b^
Rapa	(R)		E	(R)^a^	R	R		
16 °C	E		E	E	R	R		
37 °C	R^a^			ND	R	R	R^a^	
TBZ	R^a^		(R)	(R)^a^	R			

E, Epistasis is given when sensitivity of a double mutant was comparable to that of the most sensitive single mutant.

R, Rescue. (R), weak rescue effect. ^a^Rescue of *elp3Δ* phenotype. ^b^Rescue of *rpl42-P56Q* phenotype. S, Synergistic or additive effect, i.e. the *dph3Δ atf1Δ* double mutant was clearly more sensitive than the single mutants. ND, not determined.

Our study revealed that the *dph1Δ*, *dph3Δ* and *dph4Δ* mutants, but not *dph2Δ*, *dph6Δ* or *dph7Δ*, were sensitive to HU and MMS (Fig. 1 and Table 2). Thus, either Dph1 and Dph4 have additional functions apart from the eEF2 modification, or loss of the other Dph factors can be compensated. Interestingly, Dph1, Dph3 and Dph4 act in the first step of the diphthamide synthesis, whereas Dph6 and Dph7 are required for late steps^1, 5–7^. Thus, it could be that eEF2 containing intermediates of diphthamide is still functional with respect to accurate DNA damage responses, whereas unmodified eEF2 is not. Among the mutants tested, *dph3Δ* and *elp3Δ* showed the broadest phenotypic spectra (Table 2). These mutants were sensitive to low temperature, MMS, and possibly rapamycin in an epistatic relationship (Fig. 2 and Table 3). Such phenotypes were complemented by elevated tRNA^Lys^_UUU_ levels (Fig. 4), indicating that lack of the mcm^5^U_34_ modification of this tRNA species caused sensitivity. However, in the case of MMS, also *dph1Δ* was sensitive, suggesting that this particular phenotype was either partially due to unmodified eEF2, or that Dph1 has a function in tRNA modifications. Indeed, we have found that *dph1Δ* and *elp3Δ* were epistatic for MMS sensitivity (Fig. 2), supporting the latter possibility.

The *rpl42-P56Q* mutation likely affects accurate and efficient interactions between tRNAs and ribosomes, a defect that is supposed to be also caused by unmodified tRNAs and eEF2^7, 27, 31, 33, 34^. The *rpl42-P56Q* mutant was sensitive to MMS, which was partially suppressed by additional *dph3Δ* and *elp3Δ* deletions (Fig. 5). This may occur by slowing down translation due to lack of tRNA modifications, which compensates for impaired interactions between tRNAs and ribosomes caused by *rpl42-P56Q*. The *elp3Δ* mutation did not cause HU sensitivity, but allowed rescue of the HU sensitivity of *rpl42-P56Q*. *dph3Δ* was epistatic with *dph1Δ* for HU sensitivity, which was not suppressed by elevated tRNA^Lys^_UUU_ levels (Table 3). Taken together, HU sensitivity is likely due to unmodified eEF2, which appears to affect translation in a similar way as the *rpl42-P56Q* mutation. Furthermore, we would like to point out that in certain cases the *rpl42-P56Q* mutation may not be suitable for genetic screens with *S. pombe* gene deletion libraries, since *rpl42-P56Q* itself is sensitive to MMS and HU and is able to rescue temperature sensitivity.

Although *dph3Δ* and *elp3Δ* shared most of their phenotypes, we found that *elp3Δ* was extreme sensitive to 37 °C and strongly affected by TBZ, whereas *dph3Δ* exhibited minor defects under such conditions. These were more pronounced on minimal medium, indicating a link between Dph3-dependent translational control and cellular metabolisms. It has been recently shown that lack of ncm^5^U_34_ and mcm^5^U_34_ tRNA modifications in *elp3Δ* mutants cause quantitative alterations in a wide range of metabolites^35^. It is therefore possible that different metabolic states in dependency of the growth media influenced the phenotypes of *dph3Δ*. Growth inhibition and septum formation of *elp3Δ* cells at 37 °C and TBZ sensitivity were suppressed by elevated tRNA^Lys^_UUU_ levels, and also by *dph3Δ* and *dph1Δ*. We noticed that *dph3Δ* rescued growth of *elp3Δ* at 37 °C less than *dph1Δ* did (Fig. 2). This is likely not due to tRNA modification defects caused by *dph3Δ*, since the *dph3Δ dph1Δ elp3Δ* triple mutant showed full suppression. Increased tRNA^Lys^_UUU_ levels and *dph3Δ* independently suppressed growth inhibition at 37 °C and TBZ sensitivity of *elp3Δ* (Fig. 4), indicating that the *dph3Δ*-dependent rescue effect was not due to unmodified tRNA^Lys^_UUU_. We conclude that lack of the eEF2 modification caused by *dph1Δ* and *dph3Δ* compensates for lack of tRNA modifications caused by *elp3Δ*.

A recent study revealed that *S. pombe elp3Δ* mutants were H_2_O_2_ sensitive and defective in uridine wobble modification of tRNA^Lys^_UUU_, tRNA^Gln^_UUG_ and tRNA^Glu^_UUC_, but were only mildly affected in transcription of H_2_O_2_–inducible genes^25^. The H_2_O_2_–inducible genes of the transcription factors Atf1 and Pcr1 contain a high number of lysine-encoding AAA versus AAG codons, when compared to highly expressed genes. Consequently, Atf1 and Pcr1 protein levels were low after H_2_O_2_ induced stress in an *elp3Δ* background, indicating inefficient translation of these proteins due to lack of tRNA^Lys^_UUU_ wobble uridine modifications. In fact, overexpression of tRNA^Lys^_UUU_ rescued H_2_O_2_ sensitivity of *elp3Δ* mutants^25^. Another study revealed that Elp3 of *S. pombe* is required for efficient translation of specific sets of proteins, including proteins involved in cell division control, as for example the kinase Cdr2, regulating the G2/M transition of the cell cycle^26^. Importantly, elevated tRNA^Lys^_UUU_ levels rescued the growth defect of an *elp3Δ ctu1Δ* double mutant at 36 °C. Ctu1 is the homologue of *S. cerevisiae* Ncs6, which is involved in thiolation of wobble uridines in tRNAs for lysine, glutamine and glutamic acid; the same tRNAs that are modified by Elongator^16^. In our study, we found that among the mutants tested, *elp3Δ* showed the most prominent phenotypes, which were all rescued by elevated tRNA^Lys^_UUU_ levels (Table 3). Elevated tRNA^Lys^_UUU_ levels also suppressed *dph3Δ* phenotypes, with the exceptions of HU and TBZ sensitivity. Thus, the *elp3Δ* phenotypes and some of the *dph3Δ* phenotypes were at least to some extent caused by lack of the tRNA^Lys^_UUU_ modifications. This likely resulted in inefficient biosynthesis of proteins required for stress responses as it has been reported for Cdr2, Atf1 and Pcr1 in an *elp3Δ* background^25, 26^. Our data support the previous studies in that an excess of unmodified tRNA^Lys^_UUU_ can compensate for inefficient interactions with mRNAs. Importantly, we demonstrated that *elp3Δ* was epistatic with *atf1Δ* and *sty1Δ* for MMS and for growth inhibition at 16 °C, and showed positive genetic interactions (a mild rescue by *atf1Δ* and *sty1Δ*) for TBZ and rapamycin (Fig. 6). Thus, it is apparent that sensitivity of *elp3Δ* under these stress conditions was -at least partially- due to low levels of Atf1. As a consequence, transcriptional activation of stress response genes by the Atf1 transcription factor is affected. We further observed epistasis between *dph3Δ* and *atf1Δ* for rapamycin sensitivity and for growth inhibition at 16 °C and a mild rescue effect for TBZ, indicating that Dph3 ensures high Atf1 levels under such stress conditions. Although Atf1 acts as a heterodimer with Pcr1^32^, we have found that *atf1Δ* was sensitive to all stress conditions we have tested, except for growth at 37 °C, whereas *pcr1Δ* was only inhibited for growth at low and high temperatures (Fig. 6). This is consistent with a previous study, which showed that *atf1Δ*, but not *pcr1Δ*, was sensitive to salt and osmotic stress, that Atf1 bound to some promoters in the absence of Pcr1, and that induction of some genes in response to high KCl concentrations required Atf1 but not Pcr1^36^. Thus, the *elp3Δ* and *dph3Δ* phenotypes caused by low Atf1 levels, were likely due to loss of Pcr1-independent functions of Atf1 in regulating transcription of stress response genes.

Sensitivity of *elp3Δ* cells to high temperature may reflect an exacerbated situation, where not only lack of tRNA modification but also heat induced stress may affect protein biosynthesis, as it has been proposed to be the case for heat shock^37, 38^, or that protein homeostasis and/or folding is impaired at critical temperatures. It is known that in response to heat, the two subunits Ncs2 and Ncs6 of the Urm1 complex are downregulated through proteosomal degradation, which in turn affects thiolation of tRNAs and leads to altered protein levels^39^.

Defective tRNA modifications in humans have been associated with various diseases, including neurological and respiratory diseases, diabetes and different types of cancer^29^. For example, the human tRNA methyltransferase hTRM9L, which is required for methylating wobble uridines from cm^5^U_34_ to mcm^5^U_34_, is downregulated in various forms of carcinomas^40^. Re-expression in colon carcinoma cell lines allows suppression of tumour growth. Another human tRNA methyltransferase, hABH8 is required for resistance to the DNA damaging agents MMS and bleomycin^41^. Our data indicate that Elongator mediated tRNA wobble modifications and Dph catalysed diphthamide modification of eEF2 contribute to genome stability and stress responses in *S. pombe*. This is probably mediated by ensuring efficient translation of mRNAs to proteins that are required for the response to cytotoxic agents (Fig. 7). Some of the drugs we have tested here in relation to the status of *elp3* and *dph* genes are frequently used in cancer chemotherapy. Mutations in *DPH* and *ELP* genes in humans may therefore serve as prognostic markers for the outcome of chemotherapy. In addition, drugs targeting DPH and ELP proteins may be developed. In combination with chemotherapeutic agents, this would allow improved growth inhibition of cancer cells.

## Materials and Methods

### Yeast media and genetic methods

Standard yeast genetic methods and media were as described elsewhere^42^. Yeast media were YEA (yeast extract agar), YEL (yeast extract liquid), MEA (malt extract agar), MMA (minimal medium agar), and EMM (Edinburgh minimal medium). YEA contained 0.5% yeast extract, 3% glucose, 1.6% agar. YEL had the same composition as YEA, but without agar. MEA contained 3% malt extract and 1.5% agar. MMA consisted of 0.17% yeast nitrogen base without amino acids/ammonium sulphate/thiamine, 0.5% ammonium sulphate, 1% glucose, and 1.8% granulated agar. EMM was prepared using 2.73% EMM broth without nitrogen, 0.5% ammonium chloride, and 2.5% granulated agar. The supplements adenine, histidine, uracil, and leucine were added at concentrations of 100 mg/L to complex media and MEA, and if required to MMA and EMM. 5 μg/mL thiamine was added to MMA and EMM if needed. G418 (100 mg/L), or hygromycin B (200 mg/L) were added to YEA after autoclaving and cooling down of the media where required. Yeast transformation was carried out by a modified protocol of Ito *et al*.^43^, omitting the use of potentially mutagenic carrier DNA. Crosses were performed by mixing *h* ^*−*^ with *h* ^+^ strains on the sporulation medium MEA, followed by incubation for 2–3 days at 30 °C. Suspensions of cells and spore-containing asci were subsequently treated for 30 min with 30% ethanol, which selectively kills vegetative cells, and then streaked on appropriate media. After growth, colonies of the progeny were transferred to master plates and genotypes of the strains were determined after replica plating on diagnostic media. Cells were analysed with an Axioskop 2 plus microscope (Zeiss) for taking images and otherwise with a Novex B microscope (euromex). The frequency of septated cells was calculated from at least three different counts (except for data shown in Supplementary Table S1), each including 100 or 200 cells.

### Yeast strains

Yeast strains are listed in Supplementary Table S2. For strains used for spot tests or for counting septated cells, usually a second independently isolated strain with the same genotype (except possibly with the opposite mating type) was analysed. Some of those strains were not listed in Supplementary Table S2.

Strains SPAC8F11.02c (*dph3Δ*), SPAC21E11.03c (*pcr1*Δ), SPBC3B8.05 (*dph1Δ*), SPBC17D1.02 (*dph2Δ*), SPAC926.05 (*dph4Δ*), SPBC577.12 (*dph6Δ*), SPCC18.15 (*dph7Δ*), and SPAC29A4.20 (*elp3Δ*) were all from the Bioneer library^44^ version 2. The specified loci were disrupted by *kanMX*; the genetic background is *h* ^+^ *ade6* (either allele *M210* or *M216*) *leu1-32 ura4-D18*. An unrelated gene (SPAC26A3.16) has been previously named *dph1* (http://www.pombase.org/)^45^. However, for simplification and consistency with the nomenclature for other organisms, we used here the gene name *dph1* for locus SPBC3B8.05. Gene disruptions were confirmed by PCR. Also the *sty1::ura4* gene disruption was verified by PCR using primers sty1-cp5 5′-TCTGGACAACAAAGCCTTGGTAAGA-3′, ura4_cPCR_For 5′-TTATAGATAAACACCTTGGG-3′ and ura4_R1 5′-TAAAGCAAGGGCATTAAGGC-3′ in a single reaction.

The *elp3::hphMX* strain DE33 was obtained by transformation of DE23 with a *hphMX* cassette containing PCR fragment amplified with primers Ptef-F 5′-CGCCAGCTGAAGCTTCGTAC-3′ and Ttef-R 5′-GGCCACTAGTGGATCTGATA-3′ using pFA6a-hphMX6 as template^46^.

### Drug sensitivity tests

Drug sensitivities were analysed by spot tests as described previously^30^, except that we used here for most spot tests a pin multi replicator for spotting the different cell dilutions. Images were taken with a Gel Doc 2000 system (Bio-Rad) after two to three days of incubation at 30 °C, three days at 37 °C, or ten to eleven days at 16 °C. Drugs used in this study were hydroxyurea (Formedium), methyl methanesulfonate (ACROS Organics), thiabendazole (SIGMA) and rapamycin (LC Laboratories). 5 μg/mL thiamine was added to minimal medium used for spot tests with plasmid containing strains to suppress *nmt* promotor activity of pREP1.

### DAPI staining

Cells were fixed overnight in 70% ethanol, spun down and washed with phosphate-buffered saline, spun down again and then dissolved in phosphate-buffered saline containing 4′,6-diamidino-2-phenylindole (1 μg/mL).

### Cloning of *SPBTRNALYS.06*

The tRNA^Lys^_UUU_ encoding gene *SPBTRNALYS.06* together with 500 base pairs of upstream and downstream sequences was cloned into pREP1^47^ following the procedure described by Fernández-Vázquez *et al*.^25^. In brief, the gene was amplified by PCR from genomic DNA with primers tRNALys-F 5′-ACTCTGCAGATGTCAAACAGTGGCAGATG-3′ and tRNALys-R 5′-TGAGAGCTCTTTCATACTTCTTTCGCCTC-3′, digested with *Pst*I and *Sac*I and ligated with pREP1, cut by the same restriction enzymes.

### Plasmid loss of transformants

For loss of plasmids of transformed strains, 10 mL YEL was incubated with a loop-full of cell material and incubated on a shaker for two days at 30 °C. Aliquots were streaked on YEA and after growth, single colonies were tested for leucine auxotrophy.

### Preparation and electrophoresis of protein extracts

*S. pombe* strains RO144 (wild type), DE4 (*dph3Δ*), DE23 (*elp3Δ*), and DE25 (*dph3Δ elp3Δ*) were grown in 20 mL YEL at 30 °C to a density of OD_595nm_ of approximately 1. Aliquots of cells of the equivalent of 5 OD units were harvested by centrifugation, washed with 1 mL H_2_O and cell pellets were shock-frozen in liquid nitrogen and stored at −80 °C. The remaining cultures were shifted to 37 °C and aliquots were harvested after 30 min and after two hours, washed, frozen in liquid nitrogen and stored at −80 °C. 300 μL buffer H [25 mM HEPES (pH 7.5), 150 mM NaCl, 1 mM EDTA, 1 mM DTT, 0.1 mM phenylmethylsulfonyl fluoride, 5 mM β-mercaptoethanol, cOmplete EDTA-free protease inhibitor (Roche)] was added to the frozen cell pellets, which were then thawed on ice water. Cells were transferred to 2-mL screw cap tubes, mixed with 300 μL glass beads and were lysed by ten cycles of 20 sec at 6 meters/sec in a FastPrep-24 5 G (MP Biomedicals), with cooling on ice between each round. Samples were centrifuged for 15 min at 500 rpm at 4 °C to remove cell debris and intact cells. Lysates were centrifuged for 25 min at 14,000 rpm at 4 °C. Supernatants (soluble proteins) were kept on ice and pellets were suspended in buffer H, centrifuged for 20 min at 12,200 rpm at 4 °C and the resulting pellets (insoluble and chromatin bound proteins) suspended in buffer H in a volume equivalent to two thirds of the volume of the respective supernatant (approximately 120 μL). Aliquots of the fractions of soluble and insoluble proteins were boiled for 10 min at 96 °C in SDS-loading buffer and loaded on 14% SDS-polyacrylamide gels. After electrophoresis, gels were stained with InstantBlue (Expedeon). Gels were subsequently scanned and protein bands quantified with ImageJ software (NIH).

## Electronic supplementary material

Supplementary Information
