# Supplementary material for: Elp3 and Dph3 of Schizosaccharomyces pombe mediate cellular stress responses through tRNALysUUU modifications
Source: Sci Rep. 2017 Aug 3;7:7225. doi: 10.1038/s41598-017-07647-1 (PMC5543170; doi:10.1038/s41598-017-07647-1)
Supplement: Supplementary file 1 — Supplementary Information [file 41598_2017_7647_MOESM1_ESM.pdf]

Supplementary Information

Elp3 and Dph3 of *Schizosaccharomyces pombe* mediate cellular stress responses through $tRNA^{Lys}_{UUU}$ modifications

Desirée Villahermosa & Oliver Fleck

Supplementary Table S1. Frequent occurrence of septated *elp3Δ* cells at 37°C on minimal and complex medium.

Relevant genotype	Minimal medium			Complex medium		
	Number of septa per cell					
	0	1	≥2	0	1	≥2
Wild type	96	4	0	98	2	0
DE23 *elp3Δ*	85	14	1	69	21	10
DE94 w/o pREP1	79	16	5	67	29	4
DE95 w/o ptRNA$^{Lys}_{UUU}$	83	14	3	62	36	2
DE25 *dph3Δ elp3Δ*	98	2	0	95	5	0

Numbers in percent; 200 cells were counted once for each strain and medium.

DE23 is the strain that was used for spot tests as shown in Figures 1 and 2 and for counting of septa as shown in Table 1 and Figure 3.

DE94 w/o pREP1 and DE95 w/o ptRNA$^{Lys}_{UUU}$ derived from DE94 and DE95, and have lost their plasmids after being cultured in non-selective complex medium.

Supplementary Table S2. *S. pombe* strains.

Name	Genotype	Origin
BP570	*h⁻ atf1::ura4 ade6-3005 leu1-32 ura4-D18*	Ramsay McFarlane, Bangor
BP572	*h⁻ sty1::ura4 ade6-3006 leu1-32 ura4-D18*	Ramsay McFarlane, Bangor
DE4	*h⁻ dph3::loxP-ura4-loxM ura4-D18*	Villahermosa *et al.* 2017[30]
DE5	*h⁻ dph3-ATGmut ura4-D18*	Villahermosa *et al.* 2017[30]
DE19	*smt-0 dph1::kanMX*	RO144 x SPBC3B8.05
DE20	*h⁺ dph1::kanMX*	RO144 x SPBC3B8.05
DE21	*h⁻ dph3::loxP-ura4-loxM dph1::kanMX ura4-D18*	DE4 x SPBC3B8.05
DE22	*h⁺ dph3::loxP-ura4-loxM dph1::kanMX ura4-D18*	DE4 x SPBC3B8.05
DE23	*smt-0 elp3::kanMX*	RO144 x SPAC29A4.20
DE24	*h⁺ elp3::kanMX*	RO144 x SPAC29A4.20
DE25	*h⁻ dph3::loxP-ura4-loxM elp3::kanMX ura4-D18*	DE4 x SPAC29A4.20
DE26	*h⁺ dph3::loxP-ura4-loxM elp3::kanMX ura4-D18*	DE4 x SPAC29A4.20
DE33	*smt-0 elp3::hphMX*	DE23
DE43	*h⁺ elp3::hphMX ura4-D18*	DE33 x OL2138
DE47	*h⁺ dph1::kanMX elp3::hphMX*	DE20 x DE33
DE56	*h⁺ dph3::loxP-ura4-loxM dph1::kanMX elp3::hphMX ura4-D18*	DE21 x DE43
DE62	*h⁺ leu1-32*	RO144 x OL2256
DE64	*h⁺ dph3::loxP-ura4-loxM leu1-32 ura4-D18*	DE4 x OL2256
DE66	*h⁺ elp3::kanMX leu1-32*	DE23 x OL2256

DE68	h^+ *dph3::loxP-ura4-loxM elp3::kanMX leu1-32 ura4-D18*	DE25 x OL2256
DE90	h^+ *leu1-32* +pREP1	DE62
DE91	h^+ *leu1-32* +ptRNA$^{Lys}_{UUU}$	DE62
DE92	h^+ *dph3::loxP-ura4-loxM leu1-32 ura4-D18* +pREP1	DE64
DE93	h^+ *dph3::loxP-ura4-loxM leu1-32 ura4-D18* +ptRNA$^{Lys}_{UUU}$	DE64
DE94	h^+ *elp3::kanMX leu1-32* +pREP1	DE66
DE95	h^+ *elp3::kanMX leu1-32* +ptRNA$^{Lys}_{UUU}$	DE66
DE96	h^+ *dph3::loxP-ura4-loxM elp3::kanMX leu1-32 ura4-D18* +pREP1	DE68
DE97	h^+ *dph3::loxP-ura4-loxM elp3::kanMX leu1-32 ura4-D18* +ptRNA$^{Lys}_{UUU}$	DE68
DE111	*smt-0 dph2::kanMX*	RO144 x SPBC17D1.02
DE113	*smt-0 dph4::kanMX*	RO144 x SPAC926.05c
DE115	*smt-0 dph6::kanMX*	RO144 x SPBC577.12
DE117	*smt-0 dph7::kanMX*	RO144 x SPCC18.15
DE126	h^+ *elp3::hphMX rpl42-P56Q*	DE33 x EH1079
DE128	h^+ *dph3::loxP-ura4-loxM rpl42-P56Q ura4-D18*	DE22 x EH1078
DE129	h^+ *dph3::loxP-ura4-loxM rpl42-P56Q ura4-D18*	DE22 x EH1078
DE151	h^- *dph3::loxP-ura4-loxM elp3::kanMX rpl42-P56Q ura4-D18*	DE25 x DE128
EH1078	*smt-0 mat1M-rpl42*$^+$ *rpl42-P56Q ade6 leu1-32 ura4-D18*	Edgar Hartsuiker, Bangor
EH1079	h^+ *rpl42-P56Q*	Edgar Hartsuiker, Bangor

OL2138	*h$^+$ ura4-D18*	Strain collection
OL2256	*h$^+$ leu1-32 ura4-D18*	Strain collection
OL2574	*h$^-$ dph3::loxP-ura4-loxM pcr1::kanMX ura4-D18*	DE4 x SPAC21E11.03c
OL2578	*smt-0 elp3::hphMX pcr1::kanMX*	DE33 x SPAC21E11.03c
OL2581	*h$^+$ pcr1::kanMX*	DE33 x SPAC21E11.03c
OL2587	*h$^+$ elp3::hphMX atf1::ura4 ura4-D18*	DE43 x BP570
OL2588	*h$^-$ atf1::ura4 ura4-D18*	DE43 x BP570
OL2590	*h$^-$ elp3::hphMX sty1::ura4 ura4-D18*	DE43 x BP572
OL2592	*h$^-$ sty1::ura4 ura4-D18*	DE43 x BP572
OL2594	*h$^-$ dph3::kanMX atf1::ura4 ura4-D18*	OL2588 x SPAC8F11.02c
RO144	*smt-0*	Rolf Kraehenbuehl, Bangor
SPAC8F11.02c	*h$^+$ dph3::kanMX ade6 leu1-32 ura4-D18*	Kim *et al.* 2010[44]
SPAC21E11.03c	*h$^+$ pcr1::kanMX ade6 leu1-32 ura4-D18*	Kim *et al.* 2010[44]
SPAC29A4.20	*h$^+$ elp3::kanMX ade6 leu1-32 ura4-D18*	Kim *et al.* 2010[44]
SPAC926.05c	*h$^+$ dph4::kanMX ade6 leu1-32 ura4-D18*	Kim *et al.* 2010[44]
SPBC3B8.05	*h$^+$ dph1::kanMX ade6 leu1-32 ura4-D18*	Kim *et al.* 2010[44]
SPBC17D1.02	*h$^+$ dph2::kanMX ade6 leu1-32 ura4-D18*	Kim *et al.* 2010[44]
SPBC577.12	*h$^+$ dph6::kanMX ade6 leu1-32 ura4-D18*	Kim *et al.* 2010[44]
SPCC18.15	*h$^+$ dph7::kanMX ade6 leu1-32 ura4-D18*	Kim *et al.* 2010[44]

Supplementary Figure S1 Fleck

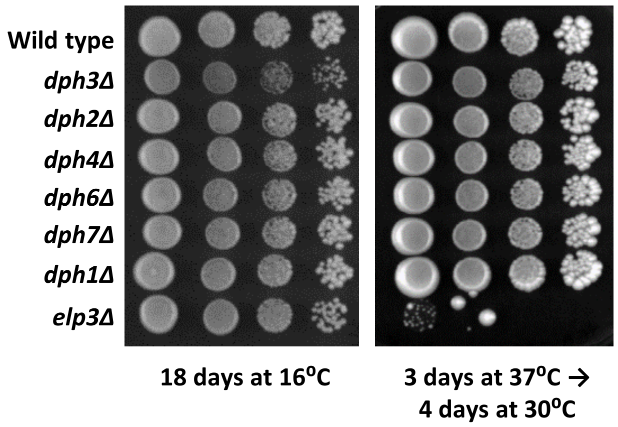

Supplementary Figure S1. Growth of *dph3Δ* and *elp3Δ* is delayed at 16°C (left). *elp3Δ* cannot recover growth when shifted from 37°C to 30°C (right). After ten days of incubation at 16°C, both *dph3Δ* and *elp3Δ* strains showed poor growth (Fig. 1); another eight days allowed the mutants to catch up in growth with wild type. Strains with the indicated genotypes were spotted on YEA in 1:10 serial dilutions and incubated for the indicated times and temperatures.

Supplementary Figure S2 Fleck

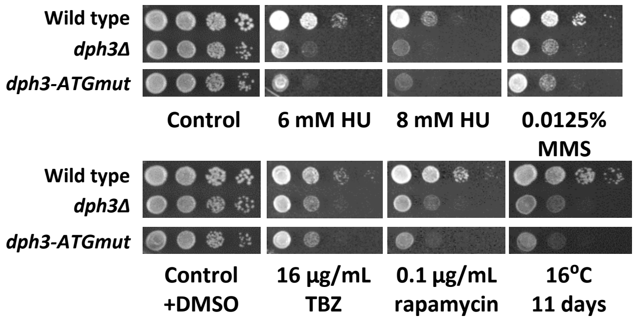

Supplementary Figure S2. A defective *dph3* gene is responsible for drug sensitivity. A *dph3-ATGmut* strain was sensitive to the indicated drugs and showed slow growth at 16ºC like *dph3Δ*. *dph3-ATGmut* has a mutated start codon and no cassette integrated that may interfere with the function of *msh3*, which is located upstream of *dph3*. Thus, the observed phenotypes were due to a defective *dph3*.

Supplementary Figure S3 Fleck

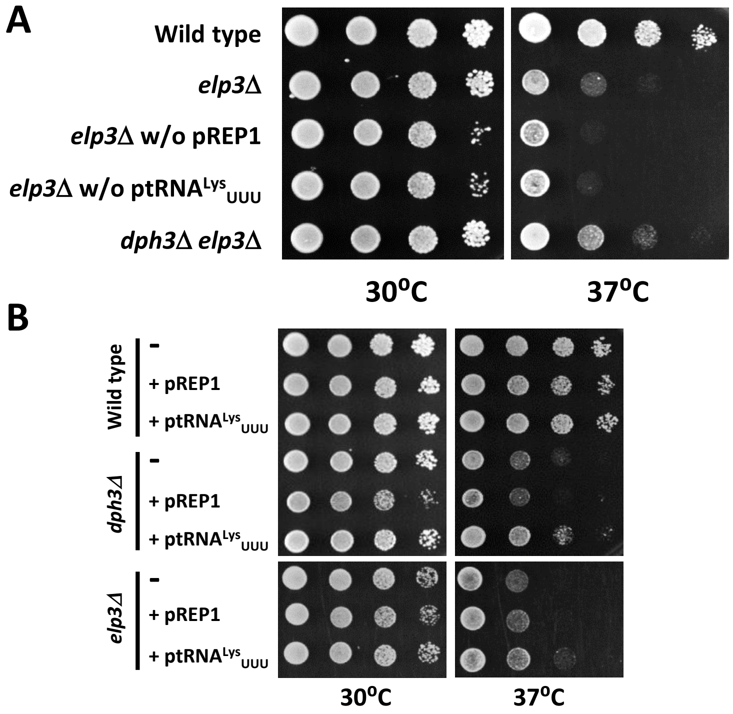

Supplementary Figure S3. Control experiments to show that elevated tRNA$^{Lys}_{UUU}$ levels were responsible for suppression of phenotypes.
(**A**) Loss of the ptRNA$^{Lys}_{UUU}$ plasmid rendered *elp3Δ* cells sensitive to 37°C. Strains were spotted on minimal medium, which allowed better growth of *elp3Δ* at 37°C than complex medium (Figs. 1 and 4C). (**B**) *dph3Δ* and *elp3Δ s*trains containing the vector pREP1 grew to the same extent at 37°C as untransformed mutant strains.

Supplementary Figure S4 Fleck

A Supernatant

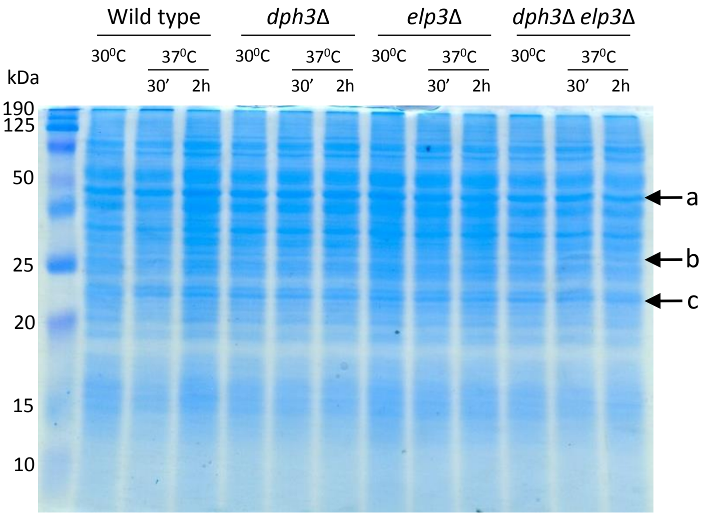

**a**	1	1	1	1	1	0.9	1.1	1	1	1	1	0.8
**b**	1	1	1	1	1	0.9	1.1	1.1	1	1	1	0.8
**c**	1	1	1	1	1	0.9	1.1	1	0.9	1	1	0.8

B Pellet

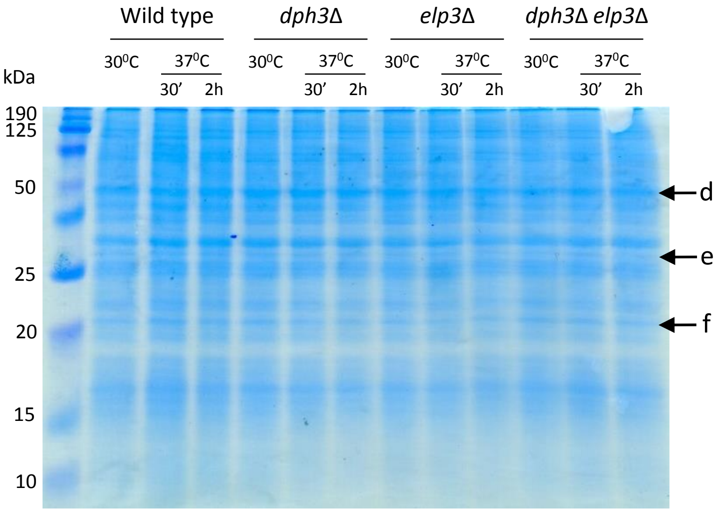

**d**	1	1	1	1.1	1	1	1.1	1	1	1	1	0.9
**e**	1	1	1	1.1	0.9	1	1	1	0.9	1.1	0.9	0.9
**f**	1	1	1	1.1	0.9	0.9	1	0.9	1	1.1	0.9	0.9

Supplementary Figure S4. Global protein levels are not significantly altered in *dph3Δ* and *elp3Δ* mutants. (**A**) Soluble protein extracts. (**B**) Insoluble and chromatin bound proteins. Protein extracts were prepared from cultures of the indicated *S. pombe* strains grown at 30⁰C and after growth for 30 min and 2 hours at 37⁰C as described in Materials and Methods. Proteins were separated on 14% SDS-polyacrylamide gels and stained with InstantBlue. Levels of proteins labelled with a to f were quantified and amounts are given below the gels relative to the protein levels of wild type grown at 30⁰C. Sizes of marker proteins are given on the left.
